# Supplementary material for: Immunodominant B-Cell Linear Epitope on the VP1 P Domain of a Feline Norovirus Cat Model
Source: Pathogens. 2022 Jun 27;11(7):731. doi: 10.3390/pathogens11070731 (PMC9316177; doi:10.3390/pathogens11070731)
Supplement: Supplementary file 1 [file pathogens-11-00731-s001.zip › pathogens-1770673-supplementary.pdf]

```
FNoV_GVI_M81          SFSVPGLIVQDMSNSRWPAQINGLVVRGNEAQVVHFQNGRCTTEGMLLGTTTLSINSIC
FNoV_GIV_CU081210E    *******V*E***********************************T*************
FNoV_GVI_M49-1        A**L*Q*T*GE********R*TTMIADPHLP**LRI******LD*T*M***Q*NP*D**

FNoV_GVI_M81          GLRGLSVS---QASVRGAPALTEEMPPLEDEVADGAAATYTLARAADTTLWLRVEEPDGR
FNoV_GIV_CU081210E    ********---*************************************************
FNoV_GVI_M49-1        RV**YFSNSRP*VMCE*EDDALDQASLGAEPIR*Q*PGLR*Q**STER--*FA*T*LN*Q

FNoV_GVI_M81          PYDIFGDQPAPLGTPDFTAVIVGTAIRPR---TTSGAYLHDAYVDTTPGDADFTPSTGNT
FNoV_GIV_CU081210E    *****************************---*A**************************
FNoV_GVI_M49-1        A**P*********V***E*R***ALV*LPGTNP**NSRFE**WI**NN-QTTYA*AA*MV

FNoV_GVI_M81          KIVLRGGGSGHVGQGHYWQFKPIAVEGGGHRPQYQEYNLPDYAGPTAS-NHDLAPPVAPR
FNoV_GIV_CU081210E    ********************R********S******************-***********
FNoV_GVI_M49-1        A*TTSDDS*SNYANDQVCE*L*LG*DITNST*-*N*FD**S*G*SAS*G*RN***T***T

FNoV_GVI_M81          MPGELLLLFESDMPVWDDGAGAAPAQKIHCLLPNEFITHLFDLQAPALAEAALLRYVHPD
FNoV_GIV_CU081210E    *****************N******************************************
FNoV_GVI_M49-1        F***IV***G*N***QNRSA*TS-NKE*R******Y*Q**Y*T***S*SDV**V**IN**

FNoV_GVI_M81          SGRTLFECKLYREGYMVVAAPAGRLNFPLDGYFRFDSWVSAFYILSPV
FNoV_GIV_CU081210E    ************************************************
FNoV_GVI_M49-1        T**V***A**H*D*F*T*N*S*T-TVL****H*******NQ**A****
```

Figure S1. The amino acid sequence alignment of the P domain of VP1 of GIV and GVI FNoVs. The asterisks indicate conserved residues. Red letters indicate the amino acid sequence matching P-10.
